# Effects of Dietary Selenium Sources on Physiological Status of Laying Hens and Production of Selenium-Enriched Eggs

**DOI:** 10.3389/fnut.2021.726770

**Published:** 2021-12-06

**Authors:** Kai Qiu, Jun-Jie Zheng, Uchechukwu Edna Obianwuna, Jing Wang, Hai-Jun Zhang, Guang-Hai Qi, Shu-Geng Wu

**Affiliations:** ^1^Risk Assessment Laboratory of Feed Derived Factors to Animal Product Quality Safety of Ministry of Agriculture & Rural Affairs, National Engineering Research Center of Biological Feed, Institute of Feed Research, Chinese Academy of Agricultural Sciences, Beijing, China; ^2^Beijing Agricultural Products Quality and Safety Center, Beijing, China

**Keywords:** Se-enriched insect protein, antioxidant capacity, egg quality, Se-enriched eggs, laying hen

## Abstract

Developing new sources of organic selenium (Se) has potential benefits for animal production and human nutrition *via* animal-based foods enriched with Se. The objective of this study was to evaluate the effects of Se-enriched insect protein (SEIP) in comparison with other sources, such as sodium selenite (SS) and selenium-enriched yeast (SEY), on performance, egg quality, selenium concentration in eggs, serum biochemical indices, immune capacity, and intestinal morphology of laying hens. Four hundred and fifty 24-week-old Hy-Line Brown laying hens with 94.0 ± 1.5% laying rate were randomly allocated to five groups with six replicates of 15 hens each. The control diet was prepared without adding exogenous selenium (calculated basal Se content of 0.08 mg/kg). The normal group was fed basal diets supplemented with 0.3 mg/kg of Se provided by sodium selenite. Three treatment groups (SS, SEY, and SEIP, respectively) were fed basal diets supplemented with 2 mg/kg of Se provided by sodium selenite, Se-enriched yeast, and SEIP, respectively. The feeding trial lasted for 12 weeks. Results revealed that dietary supplementation of 2 mg/kg of Se increased egg weight, decreased feed conversion ratio, and enhanced the antioxidant capacity of eggs in laying hens relative to the control group, whereas no significant differences were observed among SS, SEY, and SEIP treatment groups for the same. The organic source of Se provided by SEY or SEIP showed higher bio efficiency, as indicated by higher selenium content in eggs of SEY and SEIP compared with SS, although higher content was observed in SEY compared with SEIP. Also, the organic Se source significantly improved antioxidant capacity and immune functions of laying hens than the inorganic Se source. Diets supplemented with SEIP and SS significantly improved jejunal morphology of the laying hens compared with SEY, whereas SEIP was more effective than SEY to improve the oviduct health of laying hens. The results of this work evidently points the additive effect and nontoxicity of SEIP. Thus, SEIP could be used as another organic source of Se in the diet of laying hens and production of selenium-enriched eggs for humans.

## Introduction

Selenium (Se) is an essential trace element for animal and human health and plays a key role in biological functions, such as body development and metabolism, immune function, antioxidant defense system, aging, and reproduction (1–6). Nowadays, Se as a nutritional feed additive is widely used in livestock industry to maintain health and performance *via* increasing the antioxidant capacity of the animals (7, 8). As an essential mineral element, the requirement of Se for laying hens is relatively low, about 0.3 mg/kg in diets, whereas once as an nutritional additive, its supplementation should be elevated to increase bioefficiency (9). However, Se toxicosis often appears when the concentrations are slightly increased over the level as an essential element for animals (10). Therefore, it is needed to exploit low-toxic and even nontoxic Se sources for laying hens. At present, the Se additives common used in the poultry feed include inorganic forms, such as sodium selenite and nano-Se, and organic forms, like Se-enriched yeast and Se-Met (11, 12).

Optimal Se supplementation in diets is not only good for the health of laying hens but also a nutritional strategy to promote high-quality egg production (13–15). For humans, dietary Se could be used to minimize clinical complications caused by prematurity (16), but the reference values of Se intake for patients with inflammation could not be usually satisfied (17). The antagonistic action of Se on heavy metals could attenuate the adverse effects of lead and cadmium on animal health (18–22). Long-term of Se supplementation gives out a potential therapeutic effect in subjects suffering from coronary artery disease (23). Se deficiency in food inhibits myocardial development and results in hypothyroidism, a weakened immune system, Ke-shan disease, and Kaschin-beck disease (4, 24, 25). Se supplementation also plays an important role in preventing the expected metabolic alterations induced by physical inactivity and sedentary behaviors in modern life (26). Therefore, it is necessary to produce Se-enriched foods for human health.

The deposition of Se in eggs of laying hens depends on the content and source of Se in diets, and organic Se from yeast or kale sprout showed great efficiency than the inorganic Se from nano-Se or sodium selenite (27–30). In order to increase the bioefficiency of Se and its safety, Se-enriched insect protein (SEIP) was exploited through two steps of biotransformation, including microbial fermentation and insect synthesis. This work was conducted to investigate the application value of SEIP in laying hens for animal health and production of egg with high nutritional value.

## Materials and Methods

### Se Sources, Experimental Design, and Bird Management

Three kinds of Se sources were used in this work including sodium selenite, Se-enriched yeast, and SEIP. Se-enriched yeast, an inactivated Se-rich yeast product (Trademark: Alkosel) generated by the screened *Saccharomyces cerevisiae* with high Se conversion rate, was purchased from Lallemand Animal Nutrition Inc. (Montreal, Canada). Both microbial fermentation and animal biotransformation could convert inorganic Se into Se-proteins. Se conjugated to animal protein are more safe and easily digested and used by laying hens than the Se-conjugated to bacterial protein. The procedures of SEIP are described as follows. First, yeast fermentation was applied to synthesize Se-rich bacteria protein using wheat bran and soybean meal as the raw materials supplemented with sodium selenite. Then vegetables and wheat bran supplemented with the Se-rich yeast protein were supplied to yellow mealworm to obtain SEIP. Finally, the SEIP was dried and smashed into SEIP powder, which was determined to contain 4,480 mg/kg of Se and then used in the diet of laying hens.

Four hundred and fifty 24-week-old Hy-Line Brown laying hens with 94.0 ± 1.5% of egg production rate were allocated into five experimental diets in a randomized complete block design with laying rate as a blocking factor. Each group contained six replicates with 15 hens per replicate. The basal diet was formulated using specially made vitamin and mineral premix without Se supplementation, and its nutrients content (Table 1), except Se, meets or exceeds the requirements of the National Research Council (NRC, 1994) and Chinese Feeding Standard of Chicken (NY/T, 33-2004). The control group (Ctrl) was fed the basal diet. The normal group (Norm) was fed the basal diet supplemented with 0.3 mg/kg of Se provided by sodium selenite to meet the requirements of Se in NRC, 1994 and NY/T, 33-2004. To produce Se-enriched eggs and increase antioxidant capacity of laying hens, three Se treatment groups (SS, SEY, and SEIP) were fed basal diets supplemented with 2 mg/kg of Se provided by sodium selenite, Se-enriched yeast, and SEIP, respectively. The content of Se in experimental diets is shown in Table 1. The similar batches of yeast protein and insect protein without Se were used among groups to balance the nutrition contents of experimental diets.

**Table 1:** The composition and nutrient levels of experimental diets.

**Ingredient**	**Contents, %**	**Nutrients**	**Contents**
Corn	62.96	Calculated values	
Soybean meal	26.41	Crude protein, %	16.50
Limestone	8.80	Calcium, %	3.31
Dicalcium phosphate	1.00	Total phosphorus, %	0.54
Salt	0.30	Available phosphorus, %	0.33
Premix^a^	0.53	AME, MJ/kg	11.29
Total	100.00	Lysine, %	0.86
Selenium	Contents, mg/kg^b^	Methionine, %	0.37
Ctrl	0.08(0.07)	Met+Cys, %	0.65
Norm	0.38(0.33)	Analyzed values, %	
SS	2.08(1.83)	Crude protein,	16.68
SEY	2.08(1.87)	Calcium	3.37
SEIP	2.08(1.80)	Total phosphorus	0.58

a *Vitamin and mineral premix provided the following per kg of diets: VA, 12,500 IU; VD_3_, 4,125 IU; VE, 15 IU; VK, 2 mg; VB_1_, 1 mg; VB_2_, 8.5 mg; VB_6_, 8 mg; VB_12_, 5 mg; calcium pantothenate, 50 mg; niacin, 32.5 mg; biotin, 2 mg; folic acid, 5 mg; choline, 500 mg; Mn, 65 mg; I, 1 mg; Fe, 60 mg; Cu, 8 mg; Zn, 66 mg*.

b *Ctrl, the birds fed the basal diet; Norm, the birds fed 0.3 mg/kg of Se provided by sodium selenite; SS, SEY, and SEIP, the birds fed 2 mg/kg of Se provided by sodium selenite, Se yeast, and Se-enriched insect protein powder, respectively. The values out of parentheses are calculated values, and those in parentheses are analyzed values*.

Before the 12-week trial, a 1-week adaptation period was used for gradually changing the commercial diet into experimental diets. Birds had free access to water and diets throughout the experiment and were handled in accordance to Hy-Line International Online Management Guide (Hy-Line International, 2011). During the trial, all birds were raised in three-tier cages and three birds were allotted to one cage (40 × 40 × 35 cm) on 16-h of light/day with 10 −20 Lx and 14°C −20 room temperature. Egg number, egg weight, and mortality per replicate were recorded every day. Feed intake per replicate was recorded every week and adjusted once mortality occurs. Feed conversion ratio (FCR) was calculated as feed intake/egg weight (g/g).

### Sample Collection

Ten eggs per replicate were sampled at week 6 and at the end of the trial, and distributed equally for the measurement of egg quality and for the determination of cholesterol and Se contents. At week 12, the bird with similar body weight (BW) to the average BW of the replicate was selected and fasted for 12 h. After blood sampling from the wing vein, the birds were euthanized by intravenous use of pentobarbital sodium (100 mg/kg BW) and dissected under aseptic conditions. The serum was obtained by the centrifugation of the blood at 3,000 rpm/min for 15 min at 4°C and kept at −20°C until analysis. The organs or tissues were weighed including liver, heart, spleen, duodenum, jejunum, ileum, whole small intestine, oviduct, and magnum, and the value was used to calculate the relative weight of the organ or tissue to BW. After straightened, the length of the duodenum, jejunum, ileum, the whole small intestine, oviduct, and magnum were measured using a ruler. Part of jejunum was cut off gently (3 cm) and immediately fixed using 10% formalin. About 1 g oviduct tissues was sampled, homogenized in ice-bath with 2 mL PBS, and then centrifuged at 4°C and 12,000 rpm for 10 min to obtain the supernatant for ELISA analysis.

### Egg Quality Determination

The components of egg including albumen, yolk, and shell were separated and weighed for calculating their relative weight to the whole egg weight. Three points on the eggs were selected, including the air cell, equator, and sharp end, for the measurement of eggshell thickness using an Eggshell Thickness Gauge (ESTG1, Orka Technology Ltd., Ramat Hasharon, Israel). Eggshell-breaking strength was measured using an egg force reader (Orka Technology Ltd., Ramat Hasharon, Israel). An egg analyzer (Orka Technology Ltd., Ramat Hasharon, Israel) was used for the determination of albumen height, Haugh unit, and yolk color.

### Histology and Morphometric Analysis of Intestine

According to our previous report (31), intestinal villus height (VH) and crypt depth (CD) were measured by making paraffin sections of jejunum and staining with hematoxylin and eosin. The stained sections were photographed and analyzed at 40 × magnification by a microscope coupled with a digital imaging analysis system (Nikon Eclipse 80i, Nikon Co., Tokyo, Japan). For each sample, five vertically crosscutting sections were selected and 10 well-orientated villi and the corresponding crypt for one section were measured using an Image Analyzer (Lucia Software, Lucia, Za Drahou, Czechoslovakia). The VH and CD values of each sample were generated from 50 measurements. The ratio of VH and CD (V/C) was calculated.

### Chemical Analysis

All commercial kits were purchased from Nanjing Jiancheng Bioengineering Institute (Nanjing, China), and all procedures were strictly adhered to in conformation with the manufacturer's instructions. Glutamic amino transferase (ALT), aspartate amino transferase (AST), alkaline phosphatase (ALP), uric acid (UA), creatinine (CRE), and total bilirubin (TBIL) in serum was determined using the kits with catalog no. C009-2-1, C010-2-1, A059-2-1, C012-2-1, C011-2-1, and C019-1-1, respectively, on an automatic biochemical analyzer (Model 7020, Hitachi, Tokyo, Japan). The activities of glutathione peroxidase (GSH-px), superoxide dismutase (SOD), and total antioxidant capacity (T-AOC) in serum were analyzed using the kits with catalog no. A005-1-2, A001-3-2, and A015-1-2, respectively. The content of malondialdehyde (MDA) in serum and yolk was measured using the kit with catalog no A003-1-2. Contents of immunoglobulin A (IgA) and IgG in serum, and tumor necrosis factor α (TNF-α) and epidermal growth factor receptor (EGFR) in oviduct tissues were analyzed by the ELISA method using the kits with catalog no. H108, H106, H052, and H032, respectively.

According to the cholesterol (CHO) determination method (GB/T9695.24-2008) published by Standardization Administration of China, CHO content in yolk was analyzed using gas chromatography (TRACE 1300, Thermo Fisher Scientific, Rockford, IL, USA) together with an internal standard, 5α-CHO (Sigma-Aldrich, Inc., Saint Louis, MO, USA). In terms of the China National Standard (GB 5009.93-2017), the Se contents in experimental diets, whole egg, albumen, and yolk were analyzed using hydride-atomic fluorescence spectrometry (iCE 3300 AAS, Thermo Fisher Scientific, Rockford, IL, USA) coupled with a standard reference of Se (GBW8551, National Sharing Platform for Reference Materials, China).

### Statistical Analysis

All data were analyzed by one-way ANOVA procedure of SAS 9.2 (SAS Inst. Inc., Cary, NC, USA) for a completely randomized design. The value *p* ≤ 0.05 was set as the threshold for significance.

## Results

### Performance and Egg Quality

The performance indices of laying hens are shown in Table 2. The egg production during week 1–2, 3–4, 5–6, 7–8, 9–10, 11–12, and 1–12 was not influenced by the dietary treatments. The ADFI of laying hens in SEIP was lower (*p* ≤ 0.05) than other groups during week 7–8, while that during 1–2, 3–4, 5–6, 9–10, 11–12, and 1–12 was not influenced by experimental diets. During week 1–2, the laying hens in SS, SEY, and SEIP showed lower (*p* ≤ 0.05) FCR than those in control group, and the FCR of laying hens in SEIP was even smaller than those in Norm, whereas no differences were observed among the groups for FCR during week 3–4, 5–6, 7–8, 9–10, 11–12, and 1–12. The egg weight of laying hens in SEY was greater (*p* ≤ 0.05) than those in Ctrl, Norm, and SS during week 3–4. The laying hens in SS and SEY showed smaller (*p* ≤ 0.05) egg weight than those in Ctrl during week 5–6. The egg weight of laying hens in SEY was greatest (*p* ≤ 0.05) among the groups during week 9–10. There were no differences among the groups for egg weight during week 1–2, 7–8, 11–12, and 1–12.

**Table 2:** Effects of dietary Se sources on performance of laying hens.

**Items^1^**	**Ctrl**	**Norm**	**SS**	**SEY**	**SEIP**	**SEM**	***P*-value**
Egg production, %							
Week 1–2	93.23	95.76	95.16	94.05	94.2	1.05	0.48
Week 3–4	92.78	94.84	92.06	90.63	94.29	1.61	0.37
Week 5–6	88.26	88.57	90.32	92.30	91.43	2.09	0.59
Week 7–8	91.90	92.70	95.63	95.87	94.37	1.38	0.20
Week 9–10	71.51	72.94	76.03	73.02	77.22	2.81	0.59
Week 11–12	89.37	91.43	90.16	91.11	92.22	1.93	0.85
Week 1–12	88.88	90.44	90.95	90.54	91.67	0.92	0.32
ADFI, g							
Week 1–2	117.81	117.93	117.26	116.17	114.92	1.18	0.35
Week 3–4	109.36	109.03	108.37	109.73	109.22	1.15	0.94
Week 5–6	113.40	113.90	114.79	113.77	114.06	0.78	0.79
Week 7–8	116.19^ab^	116.82^a^	117.99^a^	117.79^a^	113.43^b^	1.05	0.04
Week 9–10	116.47	116.78	118.62	118.56	118.17	0.97	0.38
Week 11–12	118.03	118.04	117.18	118.90	118.31	1.23	0.90
Week 1–12	116.52	116.73	117.00	117.11	115.96	0.54	0.60
FCR, g/g							
Week 1–2	2.19^a^	2.14^ab^	2.07^bc^	2.11^bc^	2.05^c^	0.03	0.02
Week 3–4	2.01	1.97	2.01	2.04	1.96	0.04	0.55
Week 5–6	2.16	2.18	2.16	2.12	2.11	0.06	0.88
Week 7–8	2.09	2.11	2.07	2.08	2.01	0.04	0.43
Week 9–10	2.757	2.687	2.601	2.678	2.568	0.10	0.72
Week 11–12	2.179	2.127	2.135	2.124	2.114	0.04	0.77
Week 1–12	2.20	2.18	2.16	2.17	2.12	0.02	0.14
Egg weight, g							
Week 1–2	58.24	57.62	58.21	58.57	58.8	0.47	0.49
Week 3–4	58.88^b^	58.55^b^	58.46^b^	59.87^a^	59.01^ab^	0.31	0.03
Week 5–6	59.90^a^	59.01^ab^	58.95^b^	58.27^b^	59.12^ab^	0.32	0.03
Week 7–8	60.40	59.75	59.59	59.14	59.85	0.38	0.25
Week 9–10	59.73^b^	60.00^b^	60.39^ab^	60.95^a^	59.74^b^	0.30	0.04
Week 11–12	60.71	60.82	60.93	61.51	60.77	0.28	0.27
Week 1–12	59.61	59.23	59.37	59.65	59.53	0.22	0.64

a, b, c *Means within a row with no common superscripts differ significantly (p < 0.05)*.

1 *Data are the mean of eight replicates with 15 birds each; ADFI, average daily feed intake; FCR, feed conversion ratio (feed: egg, g: g); SEM, standard error of mean; Ctrl, the birds fed the basal diet; Norm, the birds fed 0.3 mg/kg of Se provided by sodium selenite; SS, SEY, and SEIP, the birds fed 2 mg/kg of Se provided by sodium selenite, Se yeast, and Se-enriched insect protein powder, respectively*.

At week 6 and 12, no differences were observed among the experimental groups for egg quality including albumen ratio, yolk ratio, shell ratio, egg shape, shell thickness, shell stiffness, shell strength, albumen height, yolk color, and haugh units (Table 3). As shown in Table 4, the Se content of the whole egg at week 6 was significantly (*p* ≤ 0.05) high with the following trend SEY>SEIP>SS>Norm>Control. At week 6, laying hens in SEY and SEIP showed lower (*p* ≤ 0.05) CHO content in yolk than those in Ctrl, and SEIP was also lower (*P* ≤ 0.05) than Norm and SS. The MDA content of yolk was significantly (*p* ≤ 0.05) decreased in turn from Ctrl, Norm, SS, SEY, to SEIP at week 6. The Se content of whole egg, albumen, and yolk at week 12 showed the same varying trend (*p* ≤ 0.05) between experimental treatments as observed at week 6. The effects of dietary treatments on the content of CHO in yolk was not kept to week 12. The MDA content of yolk in SS, SEY, and SEIP was lower (*P* ≤ 0.05) than those in SS, which is further lower (*P* ≤ 0.05) than those in Ctrl.

**Table 3:** Effects of dietary Se sources on egg quality of laying hens.

**Items^a^**	**Ctrl**	**Norm**	**SS**	**SEY**	**SEIP**	**SEM**	***P*-value**
Week 6							
Albumen ratio, %	64.70	64.90	64.59	64.91	64.29	0.28	0.52
Yolk ratio, %	24.48	24.24	24.58	24.48	24.92	0.25	0.45
Shell ratio, %	10.82	10.87	10.83	10.62	10.79	0.17	0.85
Egg shape index	1.33	1.32	1.31	1.31	1.32	0.01	0.19
Shell thickness, mm	0.455	0.447	0.448	0.453	0.453	0.01	0.90
Shell stiffness, N/mm	77.16	78.62	78.43	79.10	79.00	0.57	0.15
Shell strength, N	45.59	46.23	45.84	46.41	45.87	0.90	0.97
Albumen height, mm	7.46	7.73	7.14	7.61	7.50	0.20	0.32
Yolk color	5.58	5.63	5.53	5.43	5.43	0.15	0.84
Haugh units	86.11	87.77	83.89	87.04	86.8	1.22	0.24
Week 12							
Albumen ratio, %	64.39	64.82	64.44	64.88	64.43	0.39	0.83
Yolk ratio, %	25.33	24.82	25.25	24.76	25.36	0.36	0.64
Shell ratio, %	10.28	10.36	10.32	10.36	10.21	0.11	0.85
Egg shape index	1.32	1.31	1.32	1.31	1.32	0.01	0.39
Shell thickness, mm	0.455	0.443	0.442	0.455	0.455	0.01	0.40
Shell stiffness, N/mm	77.81	75.62	77.45	78.93	78.61	1.92	0.77
Shell strength, N	44.45	42.95	44.13	45.54	46.02	1.08	0.31
Albumen height, mm	7.39	7.75	7.51	7.67	7.91	0.15	0.15
Yolk color	7.13	7.17	7.27	7.97	7.17	0.31	0.30
Haugh units	86.13	88.04	86.77	87.79	89.17	0.94	0.21

a *Data are the mean of eight replicates; SEM, standard error of mean; Ctrl, the birds fed the basal diet; Norm, the birds fed 0.3 mg/kg of Se provided by sodium selenite; SS, SEY, and SEIP, the birds fed 2 mg/kg of Se provided by sodium selenite, Se yeast, and Se-enriched insect protein powder, respectively*.

**Table 4:** Effects of dietary Se sources on the concentration of Se, cholesterol, and malondialdehyde of eggs in laying hens.

**Items^1^**	**Ctrl**	**Norm**	**SS**	**SEY**	**SEIP**	**SEM**	***P*-value**
Week 6							
Egg- Se, μg/100 g	15.67^e^	25.67^d^	44.67^c^	115.00^a^	78.50^b^	0.86	<0.01
Yolk- CHO, mg/g	8.04^a^	7.60^ab^	7.42^ab^	7.21^bc^	6.77^c^	0.24	0.01
Yolk- MDA, nmol/mL	88.70^a^	80.12^b^	69.24^c^	72.54^cd^	67.31^d^	2.09	<0.01
Week 12							
Egg- Se, μg/100 g	15.24^e^	25.34^d^	42.26^c^	116.05^a^	83.20^b^	0.60	<0.01
Albumen- Se, μg/100 g	5.58^e^	6.92^d^	17.33^c^	98.17^a^	75.83^b^	0.42	<0.01
Yolk- Se, μg/100 g	40.33^e^	73.17^d^	107.00^c^	162.50^a^	102.33^b^	1.52	<0.01
Yolk- CHO, mg/g	7.87	7.60	7.35	6.90	7.25	0.26	0.13
Yolk- MDA, nmol/mL	85.47^a^	80.47^b^	74.33^c^	71.45^c^	72.38^c^	3.09	0.02

a, b, c *Means within a row with no common superscripts differ significantly (P < 0.05)*.

1 *Data are the mean of eight replicates; CHO, cholesterol; MDA, malondialdehyde; SEM, standard error of mean; Ctrl, the birds fed the basal diet; Norm, the birds fed 0.3 mg/kg of Se provided by sodium selenite; SS, SEY, and SEIP, the birds fed 2 mg/kg of Se provided by sodium selenite, Se yeast, and Se-enriched insect protein powder, respectively*.

### Serum Biochemistry, Antioxidant, and Immune Capacity

Biochemical indicators of serum of laying hens are listed in Table 5. At week 6, all biochemical indices of serum were not affected by experimental treatments including the activities of ALT, AST, and ALP, and the contents of UA, CRE, and TBIL. At week 12, the UA content in the serum of laying hens in Norm, SS, SEY, and SEIP was lower (*p* ≤ 0.05) than those in Ctrl. There were no differences between groups for the content of CRE and activities of ALT, AST, and ALP in serum. The laying hens in SEY had higher (*p* ≤ 0.05) TBIL content in serum than those in Ctrl, Norm, and SEIP. The TBIL content in serum of laying hens in SEIP was lower (*p* ≤ 0.05) than those in SS and SEY.

**Table 5:** Effects of dietary Se sources on blood biochemical indicators of laying hens.

**Items^1^**	**Ctrl**	**Norm**	**SS**	**SEY**	**SEIP**	**SEM**	***P*-value**
Week 6							
ALT, U/L	6.81	6.57	7.04	6.61	6.93	0.17	0.27
AST, U/L	125.66	117.21	115.50	117.28	118.20	2.60	0.08
ALP, U/L	343.68	338.74	333.20	364.94	339.08	13.57	0.52
UA, μmol/L	161.44	154.44	152.44	156.13	154.07	3.08	0.31
CRE, μmol/L	0.35	0.30	0.35	0.30	0.38	0.03	0.13
TBIL, μmol/L	17.94	17.41	18.19	17.85	17.78	0.44	0.80
Week 12							
ALT, U/L	6.42	6.65	6.48	6.61	6.76	0.20	0.77
AST, U/L	118.35	119.77	117.90	117.42	118.69	0.82	0.34
ALP, U/L	355.52	362.79	354.24	373.16	346.37	11.04	0.52
UA, μmol/L	160.46^a^	156.61^b^	155.08^b^	154.46^b^	154.49^b^	1.49	0.04
CRE, μmol/L	0.35	0.35	0.34	0.35	0.34	0.01	0.97
TBIL, μmol/L	17.56^bc^	17.49^bc^	18.11^ab^	18.29^a^	17.19^c^	0.25	0.03

a, b, c *Means within a row with no common superscripts differ significantly (P < 0.05)*.

1 *Data are the mean of eight replicates; ALT, glutamic amino transferase; AST, aspartate amino transferase; ALP, alkaline phosphatase; UA, uric acid; CRE, creatinine; TBIL, total bilirubin; SEM, standard error of mean; Ctrl, the birds fed the basal diet; Norm, the birds fed 0.3 mg/kg of Se provided by sodium selenite; SS, SEY, and SEIP, the birds fed 2 mg/kg of Se provided by sodium selenite, Se yeast, and Se-enriched insect protein powder, respectively*.

The antioxidant capacity and immunity of serum are shown in Table 6. At week 6, the activities of GSH-Px, SOD, and T-AOC, and the content of MDA in serum of laying hens in SEY and SEIP were higher (*p* ≤ 0.05) than those in Ctrl, Norm, and SS. The laying hens in SS had higher (*p* ≤ 0.05) activities of SOD and T-AOC, and MDA content in serum than those in Ctrl and Norm. No differences were observed between the groups for the contents of IgG and IgA in serum. At week 12, the activity of SOD in serum of laying hens was higher (*p* ≤ 0.05) in SEY and SEIP than those in Ctrl and Norm, and those in SEIP was also higher (*p* ≤ 0.05) than those in SS. The IgG content in serum of laying hens in SEY and SEIP was (*P* ≤ 0.05) higher than those in Norm. Laying hens in SEIP also had higher (*p* ≤ 0.05) IgG content in serum than those in Ctrl and SS, and the latter was higher (*p* ≤ 0.05) than those in the Norm. There were no differences between groups for the activities of GSH-Px and T-AOC and the content of MDA.

**Table 6:** Effects of dietary Se sources on serum antioxidant and immune capacity of laying hens.

**Items^1^**	**Ctrl**	**Norm**	**SS**	**SEY**	**SEIP**	**SEM**	***P*-value**
Week 6							
GSH-Px, U/mL	744.75^b^	745.22^b^	752.80^b^	764.35^a^	766.34^a^	3.13	<0.01
SOD, U/mL	177.18^c^	181.18^c^	194.04^b^	210.52^a^	217.42^a^	3.44	<0.01
T-AOC, U/mL	9.72^c^	9.61^c^	10.10^b^	10.63^a^	10.55^a^	0.12	<0.01
MDA, nmol/mL	5.49^a^	5.32^a^	4.96^b^	4.62^c^	4.39^c^	0.11	<0.01
IgG, g/L	4.40	4.19	4.28	4.22	4.31	0.08	0.36
IgA, g/L	2.20	2.08	2.14	2.10	2.16	0.05	0.57
Week 12							
GSH-Px, U/mL	723.75	728.76	762.49	758.78	751.55	21.86	0.63
SOD, U/mL	180.01^c^	188.52^c^	193.82^bc^	209.03^ab^	216.17^a^	5.61	<0.01
T-AOC, U/mL	9.04	9.67	11.22	11.05	10.07	0.61	0.09
MDA, nmol/mL	5.06	5.04	4.92	4.81	5.34	0.18	0.30
IgG, g/L	4.38^bc^	4.18^c^	4.69^b^	4.82^ab^	5.01^a^	0.11	<0.01
IgA, g/L	2.01^c^	2.08^bc^	2.12^abc^	2.23^ab^	2.24^a^	0.06	0.04

a, b, c *Means within a row with no common superscripts differ significantly (P < 0.05)*.

1 *Data are the mean of eight replicates; GSH-Px, glutathione peroxidase; SOD, superoxide dismutase; T-AOC, total antioxidant capacity; MDA, malondialdehyde; SEM, standard error of mean; Ctrl, the birds fed the basal diet; Norm, the birds fed 0.3 mg/kg of Se provided by sodium selenite; SS, SEY, and SEIP, the birds fed 2 mg/kg of Se provided by sodium selenite, Se yeast, and Se-rich insect protein powder, respectively*.

### Organ Indexes, Jejunum Morphology, and Oviduct Health

**Figure 1:**
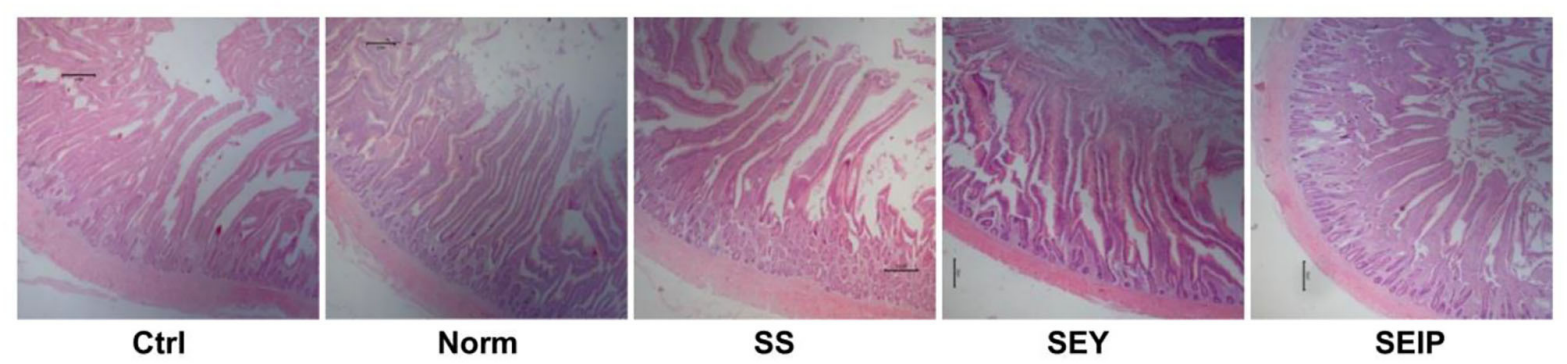
Effects of dietary Se sources on morphology of jejunum in laying hens. The representative pictures (40 ×) of jejunum section with hematoxylin and erosion (H&E) staining. Ctrl, the birds fed the basal diet; Norm, the birds fed 0.3 mg/kg of Se provided by sodium selenite; SS, SEY, and SEIP, the birds fed 2 mg/kg of Se provided by sodium selenite, Se-enriched yeast, and Se-rich insect protein powder, respectively.

As shown in Table 7, dietary treatments had no effects on the weight index of liver, heart, and spleen of laying hens. The weight and length indexes of intestine including duodenum, jejunum, ileum and the total small intestine were not changed by dietary treatments (Table 8). Figure 1 represents the sections of jejunum stained by HE, and the quantitative results of morphology are shown in Table 8. The jejunum morphology showed that the CD of jejunum of laying hens in SS and SEIP was shallower than those in Ctrl, Norm, and SEY. No differences existed between the groups for VH of jejunum. Laying hens in SS and SEIP had obvious greater (*p* ≤ 0.05) V/C of jejunum than those in Ctrl and Norm, and those in SEIP was also greater (*P* ≤ 0.05) than those in SEY. Effects of dietary treatments on oviduct health are showed in Table 9. There were no differences between the groups for the weight and length indexes of oviduct and magnum. The oviduct tissues of laying hens in Norm, SS, and SEIP showed higher (*p* ≤ 0.05) protein expression of TNF-α than those in Ctrl, and those in SEIP was also higher (*p* ≤ 0.05) than those in SEY.

**Table 7:** Effects of dietary Se sources on organ indexes of laying hens.

**Items^a^, ‰**	**Ctrl**	**Norm**	**SS**	**SEY**	**SEIP**	**SEM**	***P*-value**
Liver	18.46	18.87	18.21	18.08	18.75	0.79	0.95
Heart	3.49	3.98	3.52	3.83	3.57	0.17	0.18
Spleen	1.47	1.43	1.28	1.23	1.30	0.15	0.76

a *Data are the mean of eight replicates; The organ index was calculated as organ weight/ Live body weight. SEM, standard error of mean; Ctrl, the birds fed the basal diet; Norm, the birds fed 0.3 mg/kg of Se provided by sodium selenite; SS, SEY, and SEIP, the birds fed 2 mg/kg of Se provided by sodium selenite, Se yeast, and Se-enriched insect protein powder, respectively*.

**Table 8:** Effects of dietary Se sources on intestinal morphology of laying hens.

**Items^1^**	**Ctrl**	**Norm**	**SS**	**SEY**	**SEIP**	**SEM**	***P*-value**
Intestine index, ‰	33.15	33.28	34.35	34.32	31.51	2.09	0.87
Duodenum index, ‰	9.56	8.99	9.30	9.66	9.50	0.66	0.95
Jejunum index, ‰	13.15	13.45	14.74	12.89	11.37	1.06	0.30
Ileum index, ‰	9.04	12.53	10.31	11.76	9.67	1.06	0.88
Intestine length, mm	1,391.67	1,335.83	1,340.33	1,336.50	1,380.33	55.51	0.92
Duodenum length, mm	120.67	119.83	125.67	120.67	119.33	6.90	0.97
Jejunum length, mm	691.33	634.67	654.33	609.33	709.33	31.89	0.20
Ileum length, mm	579.67	581.33	560.33	606.50	551.67	32.33	0.78
VH, μm	1,011.34	1,049.49	959.77	1,065.56	1,127.93	55.19	0.31
CD, μm	197.33^a^	214.24^a^	159.06^b^	199.87^a^	165.53^b^	10.27	<0.01
V/C	5.15^c^	5.00^c^	6.09^ab^	5.35^bc^	6.84^a^	0.31	<0.01

a, b, c *Means within a row with no common superscripts differ significantly (P <0.05)*.

1 *Data are the mean of eight replicates; The organ index was calculated as organ weight/ Live body weight. VH, villus height of jejunum; CD, crypt depth of jejunum; V/C, the ratio of VH to CD of jejunum; SEM, standard error of mean; Ctrl, the birds fed the basal diet; Norm, the birds fed 0.3 mg/kg of Se provided by sodium selenite; SS, SEY, and SEIP, the birds fed 2 mg/kg of Se provided by sodium selenite, Se yeast, and Se-enriched insect protein powder, respectively*.

**Table 9:** Effects of dietary Se sources on the oviduct of laying hens.

**Items^1^**	**Ctrl**	**Norm**	**SS**	**SEY**	**SEIP**	**SEM**	***P*-value**
Oviduct index, ‰	32.77	31.39	35.50	35.45	34.44	1.87	0.47
Oviduct length, mm	575.00	604.67	649.33	673.00	614.00	30.60	0.21
Magnum index, ‰	14.71	14.38	15.97	15.80	14.74	1.53	0.93
Magnum length, mm	318.33	314.33	327.00	339.33	335.67	14.62	0.71
TNF-α, pg/mg	3.04^c^	3.48^ab^	3.56^ab^	3.20^bc^	3.86^a^	0.14	0.01
EGFR, ng/mg	0.69	0.72	0.73	0.71	0.77	0.02	0.25

a, b, c *Means within a row with no common superscripts differ significantly (P < 0.05)*.

1 *Data are the mean of eight replicates; The organ index was calculated as organ weight/ Live body weight. TNF-α, tumor necrosis factor α; EGFR, epidermal growth factor receptor; SEM, standard error of mean; Ctrl, the birds fed the basal diet; Norm, the birds fed 0.3 mg/kg of Se provided by sodium selenite; SS, SEY, and SEIP, the birds fed 2 mg/kg of Se provided by sodium selenite, Se yeast, and Se-enriched insect protein powder, respectively*.

## Discussion

Dietary Se supplementation is critical to maintain the performance and produce Se-enriched eggs in laying hens (15). The Se sources used in the chicken feed mainly include sodium selenite, nano-Se, Se-Met, Se-Cys, and SEY (11, 12). Se sources with high biosafety and bioefficiency need to be continuously explored and certified in the nutrition of laying hens for the production of high quality eggs. In such perspective, SEIP was produced through two steps of biotransformation from inorganic Se; both of insects and birds are animals, whereas yeast belongs to microorganism; SEIP was hypothesized to be of lower toxicity and higher bioefficiency advantages in animal feed relative to SEY. In this work, dietary supplementation of SEIP in comparison with inorganic sources SS and SEY in laying hens diets were evaluated.

Selenium, an important component of critical amino acid complexes including Se-Met and Se-Cys, plays a key role in biological processes of laying hens. Organic forms of Se are of less toxicity and have higher bioavailability and retention in tissues compared with the inorganic forms of Se (27). In this work, dietary SS, SEY, and SEIP supplementation increased egg weight and decreased FCR of laying hens relative to the control group, and no significant differences among the dietary sources were found (SS, SEY and SEIP). This is consistent with previous reports that Se supplementation culminates in significantly enhanced egg production, egg weight, daily egg mass, and feed conversion ratio compared with the control group (28, 32), but varies with the results that both source and level of SS and SEY had no effects on egg weight and FCR (29, 33, 34), and nano-Se decreased the egg production and increased the FCR (35). The variations in the studies could probably be due to different dietary levels of Se and test period because selenoprotein expression and syntheses representing bioefficiency of Se are regulated/ governed by Se level and status in the body (9).

Previous studies have demonstrated that dietary supplementation of organic or inorganic Se had no significant effect on egg quality (29, 32–34, 36) and the results from the present study confirms same. In addition, one previous study reported that the concentration of vitamin E in egg yolk was increased by dietary Se supplementation (34). For the production of Se-enriched meat and eggs, Se sources are increasingly exploited as Se supplements in animal feed. The SE from organic sources have higher bioavailability value than that from inorganic sources, which is evidenced by the Se content of eggs from birds fed the said diet (32, 37–39). It was demonstrated again by the results of this work that laying hens fed SEY or SEIP showed higher Se enriched in eggs than those of SS. This study also found that the bioefficiency of SEIP was lower relative to SEY from the Se content of eggs. High CHO content in yolk substantially constrains the consumption of eggs owing to atherosclerotic cardiovascular risk (40). The MDA which is a by-product of oxidation tends to be high in egg yolk and as such impairs the oxidative stability of fresh eggs (41). In this work, inclusion of Se in diets significantly decreased contents of CHO and MDA in yolk, which are consistent with the results of previous studies about SEY (42, 43). Therefore, it indicated that Se supplementation in diets probably contributes to enhance shelf-life of eggs besides the production of Se-enriched eggs.

The health and physiological status of the birds are often indicated by some biochemical markers. UA, an end product of purine metabolism, is often associated with multifactorial dysfunction of the kidney (44), whereas other biomarkers such as AST, ALT, ALP, and TBIL are indicators of liver inflammation and injury (45–48). The UA content in serum of laying hens was decreased by dietary Se supplementation, which is beneficial for the kidney health. The SEIP supplementation decreased the level of TBIL in the serum relative to SS and SEY in this work, which evidently points to enhanced liver function. Also, higher TBIL content in serum of birds fed SEY diet in this work was observed, but this contradicts previous reports that Se had no significant effects on serum biochemical parameters of hens and rats (33, 49) and blood clinical parameters of hens (50). Such variations may be explained by the different Se sources, inclusion levels, and animals used. GSH-Px, SOD, and catalase were considered as the first line of cell antioxidant system in birds. Se is a key element in the structure of antioxidant enzymes; therefore Se supplementation may increase the GSH-Px activity (51, 52). Diets supplemented with SS, nano-Se, or SEY improved the antioxidant balance of laying birds by increasing GSH-Px activity (28, 42, 53). Addition of Se in the diet improved antioxidant enzymes, CAT, and SOD in mice (54). The contents of T-AOC, T-SOD, and GSH-Px in the breast muscle of chicks were increased by Se supplementation (55). Se supplement enhanced the antioxidant system and immune function of laying hens *via* upregulation of GSH-Px, SOD, IgG, and IL-2 in serum (35, 56). This is consistent with the results of this work, where laying hens in the Se-diet groups showed high activity of antioxidants enzymes including GSH-Px, SOD, and T-AOC, low content of MDA, and high content of IgG and IgA in serum. This evidently points to enhanced antioxidant capacity and immune function of laying hens due to Se supplementation. The results of this work and previous works (28, 29) have demonstrated the bioavailability of organic Se in comparison with inorganic Se and efficacy to enhance immune and antioxidant system of laying hens. However, there are still some reports which showed that the effects of SS and SEY were approximately equal in promoting antioxidant capacity of laying hens (32), which is probably because of the low Se inclusion level in diets, ≤ 0.3 mg/kg.

Nano-Se supplementation has shown to improve gut function and development of broiler chickens (57, 58). In this work, laying hens fed SS and SEIP showed smaller CD and greater V/C of jejunum than those in control and SEY groups, an indication that SEIP and SS enhances intestinal absorption function of laying hens in relative to SEY. TNF-α, an inflammatory cytokine belonging to tumor necrosis factor/ tumor necrosis factor receptor (TNF/TNFR) cytokine superfamily, plays key role in biological functions, such as the maintenance of homeostatic balance and immune function, and resistance capacity to infection and cancers (59). Previous reports showed that dietary Se plays a protective role against aflatoxin B_1_ and ochratoxin A-induced inflammation and apoptosis in liver and kidney (60, 61). In this work, TNF-α expression in oviduct tissue of laying hens was significantly higher in SEIP group compared with SEY group. Thus, an efficacy of SEIP in enhancing oviduct health has been demonstrated.

## Conclusions

Diets supplemented with 2 mg/kg of inorganic or organic Se increased egg weight, decreased FCR, and enhanced the antioxidant capacity of eggs in laying hens relative to the control group, and no significant differences among the treatments SS, SEY, and SEIP was observed. The organic Se provided by SEY or SEIP showed higher bioefficiency to be deposited in eggs of laying hens relative to SS, and SEY was better than SEIP. Enhanced immune function and antioxidant capacity were associated with organic forms of Se supplementation relative to inorganic form. Diets supplemented with SEIP and SS significantly improved jejunal morphology of laying hens compared with SEY diets and SEIP significantly enhanced oviduct health relative to other diet groups. The results of this work have demonstrated the additive effect of SEIP supplementation in the production of Se-enriched eggs for humans, enhancing health and physiological status of the laying hens, and hence could be used as a feed additive of higher biosafety value than the traditional organic Se source SEY.

## Data Availability Statement

The raw data supporting the conclusions of this article will be made available by the authors, without undue reservation.

## Ethics Statement

The animal study was reviewed and approved by Animal Care and Use Committee of the Feed Research Institute of the Chinese Academy of Agricultural Sciences (Approval ID: AEC-CAAS-20191003).

## Author Contributions

S-GW, G-HQ, and H-JZ got the funding. S-GW and G-HQ conceptualized and designed the study. KQ, JW, and J-JZ performed the animal experiment. KQ, H-JZ, and UO conducted chemical analysis and analyzed the data. KQ wrote the original draft. JW, H-JZ, and UO reviewed and revised the draft. All authors agree to publish the manuscript.

## Funding

This study was supported by Beijing Innovation Consortium of Agriculture Research System (BAIC04-2021), Shandong Key Science and Technology Innovation Program (2019JZZY010704), National Natural Science Foundation of China (32072774), and Agricultural Science and Technology Innovation Program (ASTIP) of the Chinese Academy of Agricultural Sciences.

## Conflict of Interest

The authors declare that the research was conducted in the absence of any commercial or financial relationships that could be construed as a potential conflict of interest.

## Publisher's Note

All claims expressed in this article are solely those of the authors and do not necessarily represent those of their affiliated organizations, or those of the publisher, the editors and the reviewers. Any product that may be evaluated in this article, or claim that may be made by its manufacturer, is not guaranteed or endorsed by the publisher.
